# The m^6^Am methyltransferase PCIF1 promotes osteogenic differentiation of mesenchymal stem cells through stabilization of Wnt-related transcripts

**DOI:** 10.1371/journal.pbio.3003739

**Published:** 2026-04-06

**Authors:** Wei Song, Kuan-Jui Su, Zhehui Pan, Zhong Zhang, Qi Yin, Weimin Lin, Linfeng Liu, Yun Gong, Bocheng Liang, Yimeng Cai, Qiwen Li, Hui Shen, Hong-Wen Deng, Quan Yuan

**Affiliations:** 1 State Key Laboratory of Oral Diseases & National Center for Stomatology & National Clinical Research Center for Oral Diseases, West China Hospital of Stomatology, Sichuan University, Chengdu, Sichuan, China; 2 Department of Oral Implantology, West China Hospital of Stomatology, Sichuan University, Chengdu, Sichuan, China; 3 Tulane Center for Biomedical Informatics and Genomics, Deming Department of Medicine, School of Medicine, Tulane University, New Orleans, Louisiana, United States of America; 4 The Affiliated Stomatological Hospital of Nanjing Medical University & Jiangsu Province Key Laboratory of Oral Diseases & Jiangsu Province Engineering Research Center of Stomatological Translational Medicine, Nanjing Medical University, Nanjing, Jiangsu, China; Johns Hopkins University, UNITED STATES OF AMERICA

## Abstract

Osteogenesis depends on the self-renewal and differentiation of mesenchymal stem cells (MSCs). Emerging research underscores the regulatory functions of RNA methylation on bone homeostasis. Here, we show PCIF1, the N6,2′-O-dimethyladenosine (m^6^Am) methyltransferase, is essential for maintaining bone mass and promoting osteogenic differentiation of MSCs. Multiple complementary analyses—including GWAS, TWAS, and single-cell transcriptomics—collectively point to PCIF1 as a regulator of human bone mineral traits and early-stage mesenchymal differentiation. Global or MSC-specific *Pcif1* deletion elicits osteoporotic pathology in mice, although myeloid cell-specific *Pcif1* knockout does not induce femur bone alterations. Mechanistically, *Pcif1* knockout decreases m^6^Am signals of Wnt-related genes (*Wnt11*, *Fzd4*, and *Fgfr2*) and accelerates mRNA degradation. This down-regulates active β-Catenin protein, and thus impairs osteogenic function of MSCs. Additionally, the WNT agonist attenuates the osteoporosis-like phenotype induced by *Pcif1* deletion. These findings highlight the crucial role of PCIF1-mediated m^6^Am modification in regulating osteogenesis and suggest potential therapeutic implications for bone disorders.

## Abstract 2

Emerging research has highlighted the regulatory roles of m6A RNA modifications on bone homeostasis, but the function of m6Am modifications are not as well understood. This study shows that the m6Am methyltransferase PCIF1 supports bone mass maintenance by stabilizing Wnt pathway transcripts and promoting osteogenic differentiation of mesenchymal stem cells.

## Introduction

RNA epigenetics has been extensively implicated in the modulation of bone homeostasis, underscoring its pivotal role in maintaining skeletal integrity and orchestrating bone remodeling [1–5]. N6,2′-O-dimethyladenosine (m^6^Am) modification occurs on the first 2′-O-methyladenosine (Am) nucleotide at the 5′-end of capped mRNAs [6,7]. m^6^Am dynamics are synergistically regulated by the “writer” Phosphorylated CTD Interacting Factor 1 (PCIF1), the “eraser” Fat Mass and Obesity-associated Protein (FTO), and the “reader” Premature Cleavage Factor II (PCF11) [8–12]. m^6^Am modification has been closely associated with tumor cell aggressiveness, macrophage activation, and CD8^+^ T cell ferroptosis [13–17]. Currently, there is limited research on the effects of m^6^Am modification on bone health. Our team has first reported that *Pcif1* deletion could regulate murine periodontal inflammation and bone loss by inhibiting macrophage function [16]. Mesenchymal stem cell (MSC)-specific knockout of Methyltransferase like 3 (*Mettl3*), methyltransferase responsible for N6-methyladenosine (m^6^A), results in skeletal disorders and aberrant adipogenic differentiation of MSCs [18]. Furthermore, FTO, a common demethyltransferase for m^6^Am and m^6^A, is involved in regulating bone mass and osteoblast functions [19]. Given the structural similarity and functional consistency between m^6^A and m^6^Am, we propose that m^6^Am modulation has similar effects on bone development.

Bone homeostasis is primarily a dynamic balance between osteoblast-mediated bone formation and osteoclast-mediated bone resorption [20–22]. MSCs have the capacity to differentiate into osteoblasts, chondrocytes, and adipocytes in response to changes in the microenvironment and cell–cell interactions [23,24]. During osteogenesis, MSCs convert to osteoblastic progenitors, migrate to the bone surface, and participate in osteoblast maturation as well as the formation of mineralized bone tissues [25]. This process is mediated by coordinated regulation of multiple signaling pathways and transcriptional factors [26]. MSC dysfunction leads to bone developmental diseases such as osseous dysplasia, osteoporosis, and osteogenesis imperfecta [27,28]. Dissecting the regulatory molecular mechanisms of MSC biological traits could provide valuable ideas for understanding the onset and progression of skeletal diseases.

Here, we demonstrate PCIF1 is required for maintenance of bone mass and osteogenic functions of MSCs by regulating WNT signaling. Multi-omics approaches suggest *PCIF1* is associated with osteoporosis pathogenesis and might control the lineage commitment of MSCs. Whole-body or MSC-specific *Pcif1* deletion decreases trabecular bone mass and impairs osteogenic differentiation of MSCs. Mechanistically, MSC osteogenic potential is regulated through the m^6^Am-Wnt signaling, featured by the accelerated degradation rates of Wnt-related mRNA transcripts and decreased active β-Catenin level by *Pcif1* deficiency. Thus, these results highlight the significance of m^6^Am on bone formation, with potential strategies for bone repair and remodeling.

## Results

### Genetic and transcriptomic evidence links *PCIF1* to osteoporosis risk and early MSC differentiation

**Fig 1:**
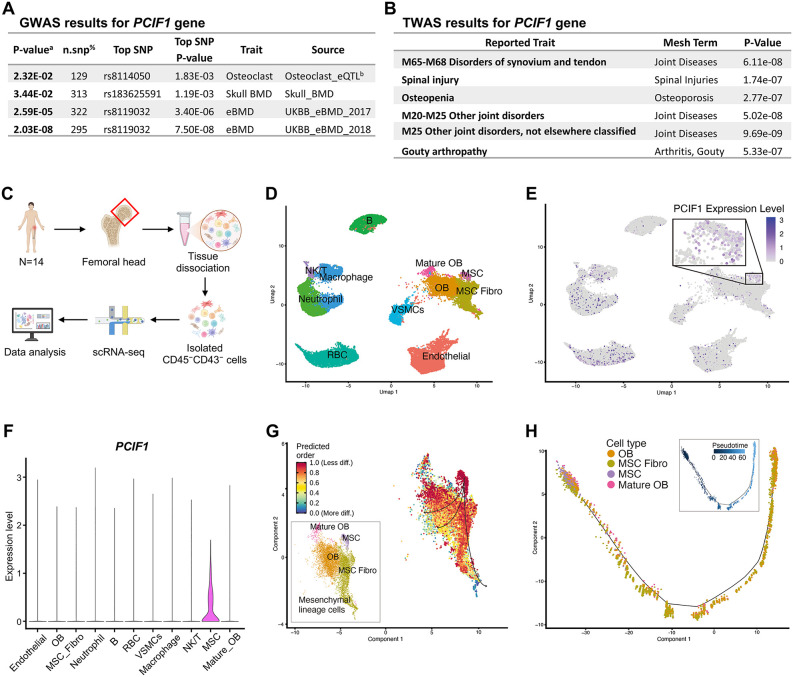
Genetic and transcriptomic evidence links *PCIF1* to osteoporosis risk and early MSC differentiation. **(A)** GWAS analysis for *PCIF1* gene. a: *P*-value for gene-based association test; b: Female samples only; %: the number of SNPs. Skull BMD: Head skull BMD; Osteoclast eQTL. TB-BMD: Total body BMD. **(B)** TWAS analysis for *PCIF1* gene identified by (Gene-based Integrative Fine-mapping through conditional TWAS; GIFT) method. **(C)** Flowchart of the scRNA-seq pipeline. This schematic diagram was Created in BioRender. Song, W. (2025) https://BioRender.com/w178r3g and *https://BioRender.com/vhpjizr*. **(D)** UMAP projection of all cell types identified from femoral head scRNA-seq data. Major immune, vascular, and skeletal cell populations were annotated, including neutrophils, macrophages, NK/T cells, endothelial cells, red blood cells (RBC), vascular smooth muscle cells (VSMCs), and stromal osteolineage populations: mesenchymal stem cells (MSC), fibroblast-like MSCs (MSC_Fibro), osteoblast precursors (OB), and mature osteoblasts (Mature OB). **(E)** *PCIF1* expression mapped onto the UMAP projection. **(F)** Violin plot showing *PCIF1* expression among human femoral head-derived bone-resident cell types. **(G)** CytoTRACE analysis of osteolineage cells. Differentiation potential predicted by CytoTRACE demonstrates a gradual loss of stemness from MSC and MSC_Fibro populations toward mature osteoblasts. **(H)** Monocle 2 pseudotime trajectory of osteoblast differentiation. Cells are arranged along a pseudotime axis showing a continuous transition from MSC_Fibro through OB to Mature OB.

To investigate the impact of *PCIF1* gene on skeletal homeostasis, we first conducted a gene-based association study involving 11 published genome-wide association studies (GWAS) focused on BMD across various skeletal sites, alongside traits such as fracture susceptibility, osteoclast activity, and estrogen levels (S1 Table). A total of 31 unique SNPs showed associations with *PCIF1* across the analyzed traits (S1 File). Four significant associations for *PCIF1* were identified in gene-based analyses of bone-related traits (Fig 1A and S1 File). The strongest associations were detected for estimated bone mineral density (eBMD) (*p* = 2.0 × 10⁻⁸, rs8119032), skull bone mineral density (BMD) (*p* = 0.034, rs183625591), and osteoclast-related eQTL traits (*p* = 0.023, rs8114050), suggesting a consistent role of PCIF1 in bone regulation.

We examined genetic associations of key m^6^Am regulators (S1 File). The m^6^Am eraser *FTO* was strongly associated with fracture risk and BMD, consistent with previous reports [19,29]. The m^6^Am reader *PCF11* also showed associations with estradiol levels and BMD. Given PCIF1’s central role as the m^6^Am writer and its impact on murine bone physiology, we therefore focused this study on elucidating PCIF1’s function in bone homeostasis.

In the transcriptome-wide association studies (TWAS), *PCIF1* was found to have 139 associations with various diseases (S3 File). Notably, it was identified as associated with osteopenia using the GIFT method (*p* = 2.77 × 10⁻⁷) and showed significant associations with M65-M68 disorders of the synovium and tendon (*p* = 6.11 × 10⁻⁸) and spinal injury (*p* = 1.74 × 10⁻⁷). Additional associations included joint disorders (*p* = 5.02 × 10⁻⁸), M25 joint disorders (*p* = 9.69 × 10⁻⁹), and gouty arthropathy (*p* = 5.33 × 10⁻⁷), suggesting a potential role for *PCIF1* in osteoporosis, joint diseases, and musculoskeletal health (Fig 1B).

To assess the cellular context and transcriptional pattern of *PCIF1* in human bone, we performed single-cell RNA sequencing (scRNA-seq) on femoral head-derived cells collected from patients undergoing total hip arthroplasty for osteoarthritis (Fig 1C). Following stringent quality control, 40,821 high-quality cells were retained for downstream analysis, with a median of 1,030 genes and 2,228 unique molecular identifiers (UMIs) detected per cell. Unsupervised graph-based clustering partitioned the transcriptomes into 11 distinct clusters (Fig 1D), encompassing both hematopoietic and non-hematopoietic populations. These included B cells, T/NK cells, neutrophils, macrophages, red blood cells (RBCs), vascular smooth muscle cells, endothelial cells, and mesenchymal lineage cells such as MSCs, fibroblast-like MSCs (MSC_Fibro), osteoblast precursors (OB), and mature osteoblasts (Mature OB). The key cell lineage marker genes are presented in S1 Fig.

Feature plot analysis revealed that *PCIF1* expression was predominantly localized to the MSC cluster, with substantially reduced expression in downstream osteolineage cells (Fig 1E and 1F). This spatial restriction highlights PCIF1 as a candidate regulator of mesenchymal stemness and suggests a potential role in maintaining progenitor identity and transcriptional readiness for osteogenic differentiation.

CytoTRACE analysis further identified MSCs as the most primitive population within the mesenchymal lineage, exhibiting the highest scores across all clusters—indicative of a stem-like, undifferentiated state (Fig 1G). MSC_Fibro cells showed intermediate scores, consistent with their known transitional and multipotent roles [30]. In contrast, OB and Mature OB populations largely displayed transcriptomic signatures of terminal differentiation, though subsets retained residual developmental plasticity.

To reconstruct the osteogenic differentiation continuum, we applied Monocle 2 pseudotime analysis, which revealed a continuous, unbranched trajectory extending from MSCs to mature osteoblasts (Fig 1H). Cells at the earliest pseudotime points overlapped with those exhibiting the highest CytoTRACE scores, reinforcing their identity as early progenitors. MSC_Fibro and OB cells occupied intermediate states, while Mature OBs were enriched at the terminus of the trajectory. Notably, *PCIF1* expression was highest at early pseudotime stages and progressively declined along the osteogenic trajectory (Fig 1H), further supporting its functional involvement in early-stage mesenchymal differentiation.

### Global deletion of *Pcif1* decreases bone mass

**Fig 2:**
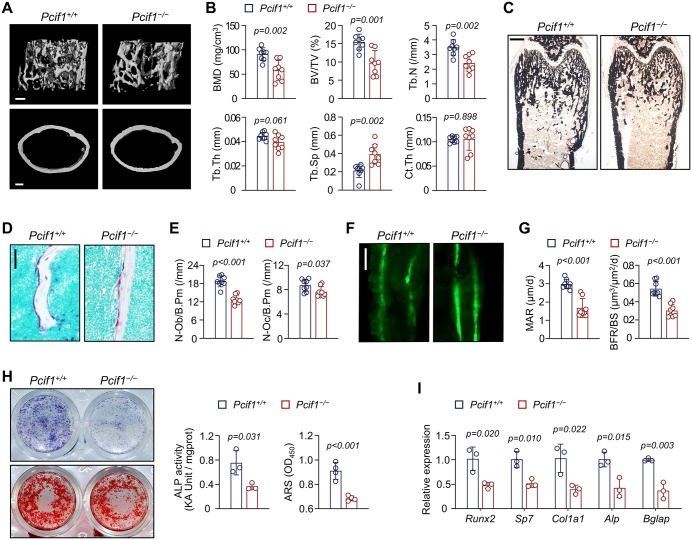
Global deletion of *Pcif1* decreases bone mass. **(A)** Representative Micro-CT images of femurs from 6-week-old male *Pcif1*^*−/−*^ knockout mice and *Pcif1*^*+/+*^ littermates. Scale bar, 200 μm. **(B)** Quantitative analyses of distal trabecular bone and midshaft cortical thickness of femurs (*n* = 8). **(C)** Representative images of Von Kossa staining of undecalcified femoral sections. Scale bar, 500 μm. **(D)** Representative TRAP images of trabecular bone from the femoral metaphysis. Scale bar, 50 μm. **(E)** Quantitation of osteoblast and osteoclast numbers of trabecular bone (*n* = 8). **(F)** Representative images demonstrating calcein double-labeling patterns in trabecular bone from the femoral metaphysis. Scale bar, 20 μm. **(G)** Quantitation of mineralization apposition rate (MAR) and bone formation rate (BFR/BS) of trabecular bone (*n* = 8). **(H)** Representative images and quantitative analyses of ALP and ARS staining of MSCs isolated from *Pcif1*^*−/−*^ knockout mice and *Pcif1*^*+/+*^ controls (*n* = 3–4). **(I)** qRT-PCR analysis of osteogenic markers in MSCs cultured with osteogenic medium for 7 days *in vitro* (*n* = 3). The data underlying panels B, E, G, H, and I can be found in S1 Data (Sheet Fig 2).

To discern the role of *Pcif1* in osteogenesis, we first assessed the bone characteristics using whole-body *Pcif1* knockout mice (*Pcif1*^*−/−*^) and control littermates (*Pcif1*^*+/+*^). Micro-computed tomography (micro-CT) analysis revealed a reduction in the trabecular bone mass of the distal femur of male *Pcif1*^*−/−*^ mice, as indicated by decreased BMD and bone volume (BV/TV) (Fig 2A and 2B). Extended research showed *Pcif1* deletion reduced trabecular number (Tb.N) and increased trabecular separation (Tb.Sp), while midshaft cortical thickness (Ct.Th) was unaffected (Fig 2B). Von Kossa staining also confirmed the impaired mineralization of distal femoral metaphysis (Fig 2C). Histomorphometric analyses suggested that *Pcif1*^*−/−*^ mice exhibited a significant reduction in osteoblast numbers (N.Ob/B.Pm) and a marginal decrease in osteoclast numbers (N.Oc/B.Pm) (Fig 2D and 2E). Loss of *Pcif1* contributed to the decreased mineral apposition rate (MAR) and bone formation rate (BFR/BS) as assessed by calcein double-labeling (Fig 2F and 2G). Female *Pcif1*^*−/−*^ mice exhibited a bone loss phenotype similar to that observed in male mice (S2 Fig).

Next, we sought to elucidate the role of *Pcif1* in osteogenic differentiation of MSCs *in vitro*. *Pcif1* deficiency markedly compromised the intensity of alkaline phosphatase (ALP) and alizarin red staining (ARS) (Fig 2H). The expression of osteogenic markers, including *Runx2*, *Sp7*, *Col1a1*, *Alp*, and *Bglap*, was also reduced (Fig 2I).

### Conditional deletion of *Pcif1* in MSCs leads to osteopenia

**Fig 3:**
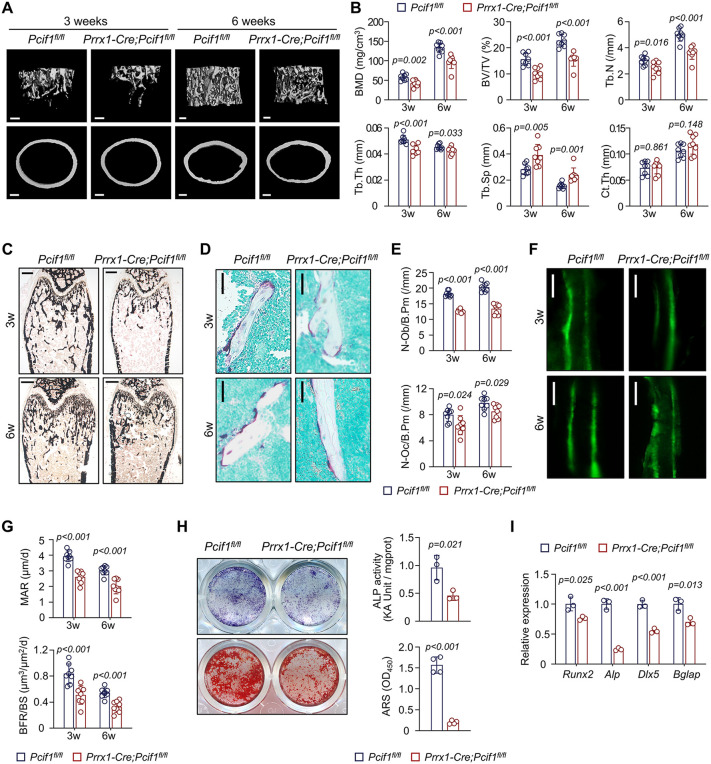
Conditional deletion of *Pcif1* in MSCs leads to osteopenia. **(A)** Representative Micro-CT images of femurs from 3-week-old or 6-week-old male *Prrx1-Cre;Pcif1*^*fl/fl*^ mice and *Pcif1*^*fl/fl*^ littermates. Scale bar, 200 μm. **(B)** Quantitative measurements of trabecular bone parameters in the distal femur and cortical bone thickness at the femoral midshaft (*n* = 8). **(C)** Representative images demonstrating Von Kossa staining of undecalcified femoral sections. Scale bar, 500 μm. **(D)** Representative TRAP images of trabecular bone at the distal end of femurs. Scale bar, 50 μm. **(E)** Quantitation of osteoblast and osteoclast numbers of trabecular bone (*n* = 8). **(F)** Representative images showing calcein double-labeling of trabecular bone. Scale bar, 20 μm. **(G)** Quantitation of MAR and BFR/BS of trabecular bone from the femoral metaphysis (*n* = 8). **(H)** Representative ALP and ARS images with quantification of MSCs isolated from *Prrx1-Cre;Pcif1*^*fl/fl*^ mice and *Pcif1*^*fl/fl*^ controls (*n* = 3–4). **(I)** qRT-PCR analysis of osteogenic gene expression in MSCs following 7 days of culture in osteogenic medium *in vitro* (*n* = 3). The data underlying panels B, E, G, H, and I can be found in S1 Data (Sheet Fig 3).

To determine whether the low bone mass of *Pcif1*^*−/−*^ mice specifically results from osteogenic defects, we generated the MSC-specific *Pcif1* knockout mice model (*Prrx1-Cre;Pcif1*^*fl/fl*^ mice). *Prrx1-Cre;Pcif1*^*fl/fl*^ mice were born alive at an expected mendelian ratio. Micro-CT revealed *Prrx1-Cre;Pcif1*^*fl/fl*^ mice had significantly reduced bone mass compared to *Pcif1*^*fl/fl*^ controls at both 3 and 6 weeks of age (Fig 3A and 3B). Quantitative results showed that ablation of *Pcif1* in *Prrx1*^*+*^ MSCs resulted in a reduction in BMD, BV/TV, Tb.N, and trabecular thickness (Tb.Th), along with an increase in Tb.Sp (Fig 3B). Ct.Th remained unchanged (Fig 3B). *Pcif1* deletion diminished the mineralized trabecular bone (Fig 3C), as well as MAR and BFR/BS of femoral trabecular bone (Fig 3F and 3G). Although the number of osteoclasts was slightly decreased, we also observed a further weakened state of osteoblasts in *Prrx1-Cre;Pcif1*^*fl/fl*^ mice (Fig 3D and 3E).

Similarly, MSCs from *Prrx1-Cre;Pcif1*^*fl/fl*^ mice showed limited osteogenic differentiation compared to *Pcif1*^*fl/fl*^ controls, as evidenced by ALP and ARS staining (Fig 3H), as well as qRT-PCR tests (Fig 3I).

### *LysM*-driven *Pcif1* knockout does not change the bone mass

**Fig 4:**
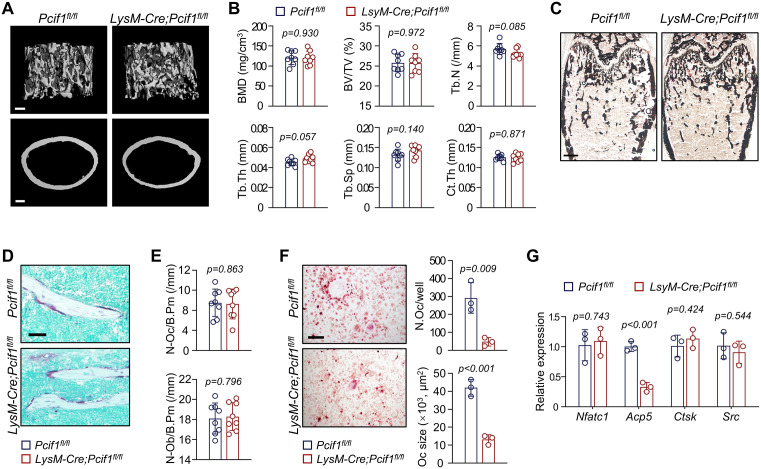
*LysM*-driven *Pcif1* knockout does not change the bone mass. **(A)** Representative Micro-CT images of femurs from 6-week-old male *LysM-Cre;Pcif1*^*fl/fl*^ mice and *Pcif1*^*fl/fl*^ littermates. Scale bar, 200 μm. **(B)** Quantitation of bone parameters (*n* = 8). **(C)** Representative Von Kossa images of undecalcified femoral sections. Scale bar, 500 μm. **(D)** Representative TRAP images of distal trabecular bone. Scale bar, 50 μm. **(E)** Quantitation of osteoclast and osteoblast numbers of trabecular bone (*n* = 8). **(F)** Representative *in-vitro* TRAP images and osteoclast quantification from BMDMs of *LysM-Cre;Pcif1*^*fl/fl*^ mice and *Pcif1*^*fl/fl*^ controls (*n* = 3). Scale bar, 200 μm. **(G)** qRT-PCR analysis of osteoclast markers *in vitro* (*n* = 3). The data underlying panels B, E, F, and G can be found in S1 Data (Sheet Fig 4).

Bone homeostasis relies on the equilibrium between osteogenesis and osteoclastogenesis [31–33]. Tartrate-resistant acid phosphatase (TRAP) staining showed reduced bone resorption occurring in femoral trabecular bone of *Pcif1*^*−/−*^ mice (Fig 2D and 2E). Although the change in osteoclast numbers was smaller than that in osteoblast numbers (Fig 2E), we investigated whether *Pcif1* deletion could directly induce osteoclast impairment *in vivo* by generating myeloid cell-specific *Pcif1* knockout mice (*LysM-Cre;Pcif1*^*fl/fl*^ mice) [16]. Femurs from 6-week-old mice exhibited comparable bone parameters between *LysM-Cre;Pcif1*^*fl/fl*^ mice and *Pcif1*^*fl/fl*^ littermates, regardless of sex, as supported by BMD, BV/TV, Tb.N, Tb.Th, Tb.Sp, and Ct.Th (Figs 4A, 4B, and S3). Histomorphometric measurements did not show notable difference by *LysM*-driven deletion of *Pcif1* (Fig 4C–4E). Although *in-vitro* induction of BMDMs derived from *LysM-Cre;Pcif1*^*fl/fl*^ mice displayed decreased osteoclast numbers and sizes (Fig 4F), the expression of osteoclast markers did not differ between two groups (Fig 4G).

### PCIF1-mediated m^6^Am modification regulates osteogenic differentiation of MSCs through WNT signaling

**Fig 5:**
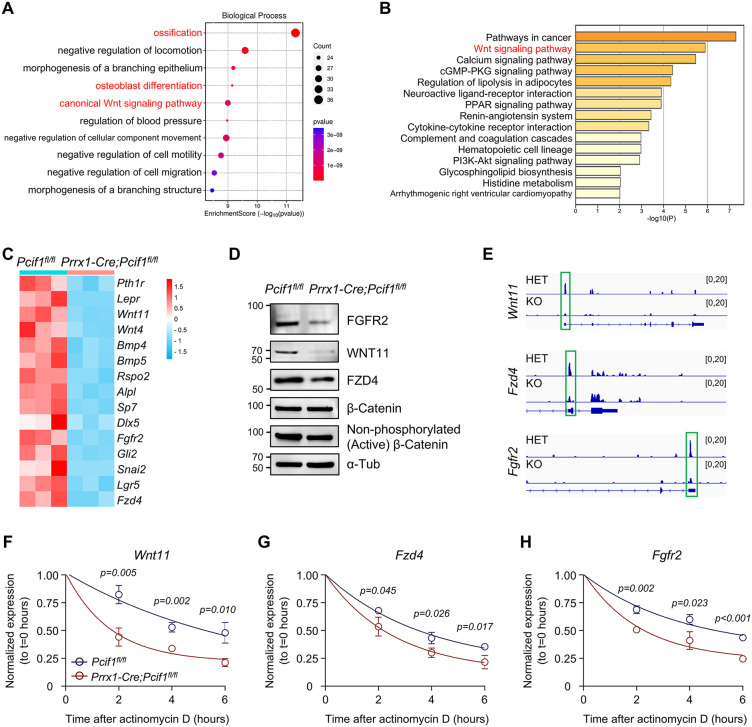
PCIF1-mediated m^6^Am modification regulates osteogenic differentiation of MSCs through WNT signaling. **(A)** GO analysis of differentially expressed genes by RNA-seq of MSCs from *Prrx1-Cre;Pcif1*^*fl/fl*^ mice and *Pcif1*^*fl/fl*^ controls after 7 days of osteogenic induction. **(B)** KEGG analysis of significantly down-regulated genes. **(C)** Heatmap of representative down-regulated genes associated with ossification and Wnt signaling. **(D)** Western blot showing expression of Wnt-related proteins. **(E)** IGV images showing down-regulated m^6^Am peaks of *Wnt11*, *Fzd4*, *and Fgfr2* mRNAs by *Pcif1* deletion. **(F–H)** RNA decay analyses of *Wnt11*, *Fzd4*, *and Fgfr2* in MSCs after 7-day osteogenic induction *in vitro* (*n* = 3). The data underlying panels F, G, and H can be found in S1 Data (Sheet Fig 5).

To uncover the potential mechanism of PCIF1 in regulating osteogenesis, we performed bulk RNA-seq using MSCs from *Prrx1-Cre;Pcif1*^*fl/fl*^ mice and *Pcif1*^*fl/fl*^ littermates after 7 days of osteoblast induction. A total of 370 down-regulated genes and 162 up-regulated genes were identified (|log_2_FoldChange| ≥ 1, padj ＜ 0.05) (S4A Fig). Gene ontology (GO) analysis revealed that ossification, osteoblast differentiation, and canonical Wnt signaling pathway were the most significantly down-regulated gene sets in *Pcif1*-deficient cells (Fig 5A). Kyoto Encyclopedia of Genes and Genomes (KEGG) assessment suggested *Pcif1* deficiency may mainly affect Wnt signaling pathway, calcium signaling pathway, and cGMP-PKG signaling pathway (Fig 5B). We found that *Pcif1* knockout reduced the expression of osteogenic marker genes, such as *Dlx5*, *Alpl*, *Sp7*, *Bmp4*, and *Bmp5*, as well as Wnt-related genes, including *Wnt11*, *Wnt4*, *Fgfr2*, *Fzd4*, *Snai2*, *Lgr5*, *Pth1r*, and *Rspo2* (Fig 5C). Western blot confirmed a decrease in WNT11, FZD4, and FGFR2 protein levels by *Pcif1* deficiency (Fig 5D). Additionally, we examined expression levels of Wnt-related transcripts in MSCs under basal conditions. *Pcif1* deletion markedly reduced *Wnt11* and *Fzd4* expression in undifferentiated MSCs, though *Fgfr2* remained unchanged (S4B Fig).

By integrating our transcriptome profiles, the m^6^Am database (GSE151229), and the Wnt signaling pathway gene set (mmu04310) (S4C Fig), we proposed that PCIF1-mediated m^6^Am modification was prevalent in the canonical and non-canonical WNT signaling pathways. Specifically, we prioritized several genes for a detailed analysis, focusing on those with m^6^Am peaks present at the transcription start sites (TSS) (Fig 5E). Wnt family member 11 (Wnt11) has been recognized as one of the predominant non-canonical WNT ligands [34,35]. Frizzled class receptor 4 (Fzd4) primarily participates in the canonical WNT signaling pathway, activating the β-Catenin-dependent signaling cascade, thereby initiating the expression of genes associated with bone development [36–38]. Fibroblast growth factor receptor 2 (Fgfr2) and the WNT signaling pathway act synergistically, particularly in skeletal and craniofacial development [39–41]. Loss of *Pcif1* decreased m^6^Am modification levels on the *Wnt11*, *Fzd4*, and *Fgfr2* mRNAs (Fig 5E). In addition, *Pcif1* knockout reduced the expression of active β-Catenin, but no significant difference was observed in β-Catenin levels (Fig 5D).

To further assess the effect of m^6^Am modulation on the aforementioned mRNA transcripts, we examined their decay rates. Following a 7-day osteoblastic induction period, *Pcif1*-deficient cells and *Pcif1*-wildtype cells were treated with actinomycin D. After 2, 4, or 6 hours of treatment, *Wnt11* mRNA in *Pcif1*-deleted cells exhibited greater degradation compared to that in the control cells (Fig 5F). The mRNA levels of *Fzd4* and *Fgfr2* exhibited similar trends (Fig 5G and 5H).

### Functional verification of candidate m^6^Am-modified genes

**Fig 6:**
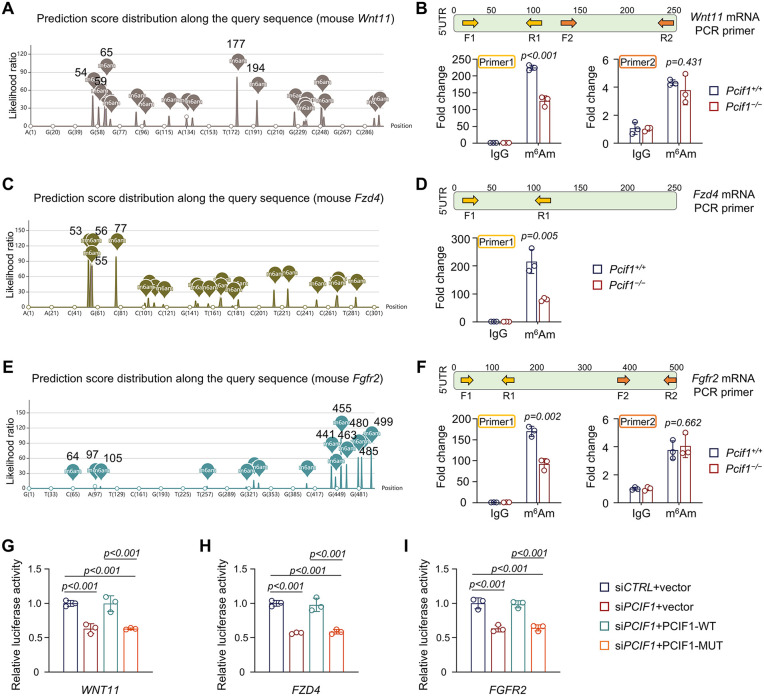
Functional verification of candidate m^6^Am-modified genes. **(A)** The calculated likelihood ratio of m^6^Am modification sites on mouse *Wnt11* mRNA predicted by m6AmPred database. **(B)** MeRIP-qPCR analysis of m^6^Am enrichment at two predicted sites within *Wnt11* mRNA in MSCs. **(C)** The calculated likelihood ratio of m^6^Am modification sites on mouse *Fzd4* mRNA predicted by m6AmPred database. **(D)** Detection of the m^6^Am level in *Fzd4* mRNA by MeRIP-qPCR. **(E)** The calculated likelihood ratio of m^6^Am modification sites on mouse *Fgfr2* mRNA predicted by m6AmPred database. **(F)** MeRIP-qPCR–based quantification of m^6^Am enrichment at predicted sites in *Fgfr2* mRNA. **(G–I)** Relative luciferase activity of *WNT11* **(G)**, *FZD4* **(H)**, and *FGFR2* (I) in 293T cells co-transfected with *PCIF1* siRNA and *PCIF1* overexpression plasmid. The data underlying panels B, D, F, G, H, and I can be found in S1 Data (Sheet Fig 6).

To examine whether these Wnt-related genes are regulated by m^6^Am modifications in MSCs, we predicted the m^6^Am distribution using the m6AmPred database [42]. Mouse *Wnt11* transcript revealed a high likelihood of m^6^Am modification within the first 200 nt downstream of the TSS, particularly at position 56, 59, 65, 177, and 194 (Fig 6A). We further performed MeRIP-qPCR to assess m^6^Am enrichment on the *Wnt11* mRNA using two primer sets (Primer 1, 0:+125 distance; Primer 2, +125:+250 distance). Primer 1 detected a prominent m^6^Am peak near the 5′-UTR, and the m^6^Am level was markedly reduced upon *Pcif1* deficiency (Fig 6B). In contrast, Primer 2 showed low enrichment in the corresponding region (Fig 6B). Collectively, these results indicated that m^6^Am modification is present on the adenosine located downstream of the *Wnt11* TSS.

As for *Fzd4*, potential m^6^Am modifications were modeled within the first 100 nucleotides (position 53, 55, 56, and 77) (Fig 6C). Similarly, by examining this region, we confirmed that loss of *Pcif1* in MSCs led to an over 50% reduction of m^6^Am level on *Fzd4* mRNA (Fig 6D). While the +400:+500 sequence of *Fgfr2* transcript was predicted to have a much higher probability than the 0:+150 region (Fig 6E), MeRIP-qPCR results revealed a completely opposite conclusion. The 0:+150 region (Primer 1), rather than the +400:+500 region (Primer 2), was identified as the m^6^Am-modified site (Fig 6F).

To directly assess the role of m^6^Am in regulating *WNT11* mRNA stability, we generated a dual-luciferase *WNT11* reporter plasmid, a wild-type PCIF1 overexpression plasmid (PCIF1-WT), and a catalytic-dead PCIF1 mutant plasmid (PCIF1-MUT) [13,15]. Overexpression of wild-type PCIF1 restored the compromised *WNT11* expression induced by *PCIF1* knockdown (Fig 6G). Catalytically inactive PCIF1 could not increase the *WNT11* level in the si*PCIF1* group (Fig 6G). Likewise, similar results were also observed for the expression of *FZD4* and *FGFR2* (Fig 6H and 6I), suggesting that the catalytic activity of PCIF1 is essential for mRNA stability of these genes.

### WNT agonist mitigates bone loss induced by *Pcif1* deletion

**Fig 7:**
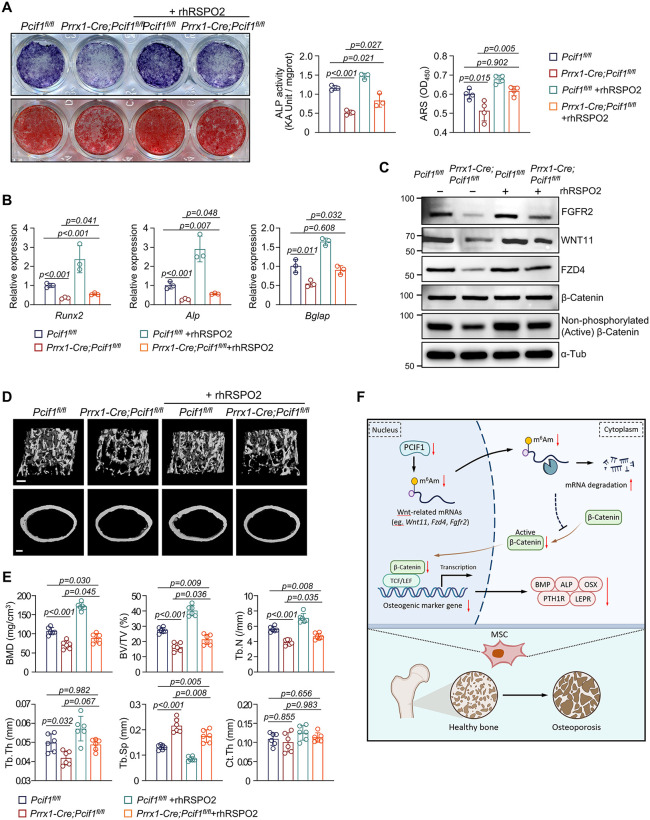
WNT agonist mitigates bone loss induced by *Pcif1* deletion. **(A)** Representative images and measurements of ALP and ARS staining of MSCs (*n* = 3–4). **(B)** qRT-PCR analysis of osteogenic markers in MSCs after induction for 7 days (*n* = 3). **(C)** Western blot showing expression of Wnt-related proteins. **(D)** Representative Micro-CT images of distal femur metaphysis and midshaft cortical bone after administration of WNT agonist. Scale bar, 200 μm. **(E)** Quantitation of bone volume (*n* = 6). **(F)** Schematic diagram of the manuscript created using selected icons from *BioRender. Song,* ***W.*** *(2025)* *https://BioRender.com/lihviy1*. The data underlying panels A, B, and E can be found in S1 Data (Sheet Fig 7).

R-spondin-2 (RSPO2) has been widely identified as an activator of canonical WNT signaling [43–46]. We treated MSCs from *Prrx1-Cre;Pcif1*^*fl/fl*^ mice and *Pcif1*^*fl/fl*^ littermates with recombinant RSPO2 (rhRSPO2) (100ng/mL). After 7 days of osteoblast induction, rhRSPO2 treatment enhanced the ALP activity and mineralization of *Pcif1*-wildtype cells (Fig 7A). In detail, rhRSPO2 partially restored the impaired osteogenic capacity of *Pcif1*-deleted cells, although the recovery did not reach the original level observed in *Pcif1*-wildtype cells (Fig 7A). qRT-PCR results showed the decreased expression of *Runx2*, *Alp*, and *Bglap* by *Pcif1* deficiency was elevated by rhRSPO2 (Fig 7B). Besides, the compromised expression of WNT11, FZD4, FGFR2, and active β-Catenin in *Pcif1*-knockout cells were up-regulated by rhRSPO2 treatment (Fig 7C).

To test the effect *in vivo*, we performed weekly intraperitoneal injection of rhRSPO2 (4 mg/kg) to 3-week-old *Prrx1-Cre;Pcif1*^*fl/fl*^ male mice for five weeks. Notably, the bone deformities caused by *Pcif1* deficiency were alleviated (Fig 7D). The trabecular parameters of *Prrx1-Cre;Pcif1*^*fl/fl*^ mice, including BMD, BV/TV, Tb.N, and Tb.Th, demonstrated notable improvement following rhRSPO2 injection (Fig 7E). As well, the increased Tb.Sp by *Pcif1* deletion was effectively ameliorated by rhRSPO2 treatment (Fig 7E).

## Discussion

Emerging evidence has elaborated the role of m^6^Am modification in tumorigenesis, anti-tumor immunity, anti-infection responses, and various biological processes [15–17,47–49]. Since PCIF1 has been identified as the methyltransferase for editing m^6^Am peaks [50,51], it has become a prominent research topic in the field of epigenetic regulation. Existing studies have mainly focuses on the immunological functions of m^6^Am modification, with limited attention given to its role in bone elongation. Moreover, m^6^A and m^6^Am exert similar functions in bone physiology, probably because of their structural similarity. Our previous work has unveiled that m^6^A methyltransferase METTL3 modulates MSC differentiation, tooth root formation, calvarial ossification, cartilage development and periodontal inflammation [2,5,18,52,53]. In contrast, current research on m^6^Am has predominantly centered on its immunological functions. Our group remains the only one to provide evidence that m^6^Am regulates periodontal bone loss, a biological effect that parallels the role of METTL3-mediated m^6^A modification in periodontal inflammation-associated bone loss. In this study, we further demonstrate that m^6^Am-Wnt signaling regulates femoral bone mass, expanding the known scope of m^6^Am function in skeleton development, and suggesting its potential as a therapeutic target for osteoporosis.

We used a combination of human gene-based association genetic studies from large population-based cohorts and scRNA-seq data to investigate the role of *PCIF1* in bone-related traits. GWAS and TWAS studies identified the strong associations between *PCIF1* and eBMD or bone disorders. Notably, to the best of our knowledge, previous genome-wide and transcriptome-wide studies have not directly implicated *PCIF1* in bone-related traits. Our current gene-based results revealed that *PCIF1* harbors multiple SNPs associated with bone phenotypes, with the lead SNP rs8119032 showing genome-wide significance (*p* = 2.0 × 10⁻⁸). Annotation of the lead *PCIF1*-associated SNPs (rs8119032, rs18362559, and rs8114050) using Ensembl showed that they lie in intronic or proximal intergenic regions relative to *PCIF1*, a genomic context where many regulatory elements reside. Although such noncoding variants frequently act through regulatory mechanisms, none of the lead SNPs appeared as significant *cis-*eQTLs for *PCIF1* in GTEx v8 single-tissue datasets. This suggests that any regulatory effects may be tissue-specific, modest in magnitude, or restricted to bone-related or disease-associated cellular contexts that are not represented in GTEx. Thus, while our genetic analyses robustly implicate the *PCIF1* locus in bone traits, the precise regulatory elements and mechanisms linking these noncoding variants to PCIF1 activity remain to be determined by future functional studies. In line with our previous report [16], *PCIF1* also showed significant correlation with osteoclasts, indicating that it may act as a broader regulator of bone homeostasis. These findings nominate *PCIF1* as a previously unrecognized genetic contributor to bone health and provide functional insight into its role.

Our scRNA-seq analysis of femoral head-derived bone-resident cells revealed that *PCIF1* is selectively expressed in MSCs and progressively declines along the osteogenic differentiation trajectory, suggesting a role in early osteolineage programming. This expression pattern implies that *PCIF1* may contribute to the maintenance of MSC identity or the regulation of early differentiation events. The use of femoral head tissue from total hip arthroplasty remains a practical and ethically appropriate approach for obtaining human bone cells. Since our scRNA-seq data were derived from femoral head tissue of patients with osteoarthritis, we acknowledge that disease-associated inflammatory factors might influence the transcriptional profiles or composition of bone-resident MSCs. Although evidence suggests that osteoarthritis has limited effects on deep trabecular bone [30], the potential impact of disease-related changes on *PCIF1* expression and MSC heterogeneity cannot be fully excluded. We note that our findings may not fully represent the transcriptional landscape of healthy bone. Nevertheless, this dataset provides a valuable foundation for future comparative studies aimed at validating PCIF1’s role in normal bone development and homeostasis. The elevated expression of *PCIF1* in undifferentiated MSCs suggests that PCIF1-mediated m^6^Am methylation may help sustain gene expression programs essential for stemness and inhibit premature lineage commitment.

Notably, cells with high *PCIF1* expression also exhibited gene expression profiles characteristic of early-stage progenitors, while more differentiated osteoblasts showed minimal *PCIF1* activity. This graded expression pattern supports the idea that PCIF1 may act as a molecular checkpoint in osteogenic differentiation, helping to regulate the transition from stemness to commitment. Similar functions for RNA methylation machinery have been described in other stem cell systems, such as the role of METTL3-mediated m^6^A methylation in promoting differentiation in embryonic stem cells [54]. While PCIF1 catalyzes the related m^6^Am modification, its expression pattern in MSCs suggests it may similarly modulate stemness and lineage progression in skeletal tissue. Our findings extend the regulatory role of RNA methylation in cell fate decisions to the context of bone development. Based on these data, we hypothesized that PCIF1 was strongly correlated with bone mass, and might regulate bone homeostasis via osteoblast–osteoclast balance.

To verify this hypothesis, we performed extensive studies using several *Pcif1*-knockout mouse models. We first observed global *Pcif1* ablation mice had shorter size and reduced trabecular bone measurements, while cortical bone thickness was unaffected by *Pcif1* deficiency. Notably, loss of *Pcif1* led to a decrease in osteoblast and osteoclast numbers, with the more pronounced downregulation of osteoblasts compared with osteoclasts. The observed reduction in osteoclasts may reflect altered RANKL/OPG signaling secondary to compromised osteoblast function, as osteoblasts are a major source of RANKL and OPG [55,56]. Hence, we introduced *Prrx1-Cre* and *LysM-Cre* for conditional *Pcif1* knockout in MSC and myeloid cells, respectively, to further explore whether PCIF1 targets at osteogenesis or osteoclastogenesis. *Prrx1*-driven *Pcif1* knockout induced delayed bone formation in murine femurs, resembling the phenotype observed in systemic knockout mice. However, *LysM*-driven *Pcif1* deletion did not cause changes of trabecular volumetric BMD, though *in-vitro* culture showed compromised osteoclast differentiation. We propose that the effects of *Pcif1* on osteoclasts may be masked or compensated by other factors *in vivo*, and unable to produce measurable changes. Moreover, osteoclast formation is mainly driven by RANKL *in vitro*, and could not fully recapitulate the complex microenvironment found *in vivo*. In this paper, we prioritized MSCs as the principal cell population affected by *Pcif1* deficiency during skeleton development.

WNT signaling facilitates MSC renewal and osteoblast differentiation and inhibits osteoblast apoptosis [57–59]. Accumulating reports have confirmed the crucial role of WNT signaling in skeletal development and bone regeneration [60–62]. Our RNA-seq of osteogenic MSCs results showed the most significant downregulation pathways including ossification and Wnt signaling. Wnt-related genes exhibited widespread downregulation, suggesting PCIF1 could broadly affect WNT pathway rather than targeting a single gene or a limited subset of genes. By combing analyses of a published m^6^Am-exo-seq database, we noticed the TSS regions of *Wnt11*, *Fzd4*, and *Fgfr2* mRNA had PCIF1-mediated m^6^Am peaks. WNT11 is a potent ligand of non-canonical WNT signaling [63,64], regulating osteoblast maturation and bone formation through RSPO2-LGR5 pathway [65,66]. Overexpression of *FZD4* restores the inhibited bone formation by mechanical unloading [67]. Inducible FGFR2 activation enhances the osteogenesis by activating Wnt/β‐catenin signaling after bone marrow ablation [68]. *FGFR2* mutations cause lethally systemic skeletal malformation in human subjects [69]. Our previous research has reported that FGFR2 ligand FGF2 controls BMSC fate by modulating rDNA chromatin architecture through phase separation [70]. Existing literature has illustrated that PCIF1-mediated m^6^Am regulation controls mRNA stability [8,14,16,47]. Here, we found *Pcif1* deficiency accelerated decay rates of *Wnt11*, *Fzd4*, and *Fgfr2* mRNAs, indicating that PCIF1 manipulates WNT signaling by stabilizing mRNAs. We validated the down-regulated expression of WNT11, FZD4, and FGFR2 proteins, as well as the decreased levels of active β-Catenin.

Given the epitranscriptomic landscape is highly cell-type and context-specific, we integrated m6AmPred predictions with MeRIP-qPCR for further validation. We subjected the *Pcif1*-deleted MSCs and WT cells to m^6^Am enrichment, and designed the primers according to the prediction scores by m6AmPred. Mouse *Wnt11* and *Fzd4* transcripts showed significant m^6^Am distribution within the first ~150 nt downstream of TSS, which were consistent with previous reports [10,51]. The pronounced loss of these signals in the *Pcif1*-deleted cells strongly supported that the detected modification corresponds to m^6^Am. Interestingly, mouse *Fgfr2* transcript got higher score in the+400:+500 region, whereas MeRIP-qPCR results identified the m^6^Am peaks within the 0:+150 region. This might be ascribed to the limitations of the prediction model, the complexity of the sequence context, and cell-type-specific TSS selection. Dual-luciferase reporter assays also showed that exogenous expression of PCIF1 with m^6^Am catalytic activity was able to restore the expression of *WNT11*, *FZD4*, and *FGFR2* in *PCIF1*-knockdown cells. A limitation is that, although m^6^Am was detected, its precise modification sites remain to be determined. Further investigations are needed to elucidate the broader landscape of m^6^Am-mediated regulation.

We next evaluated whether the WNT activator RSPO2 could function as potential therapeutic intervention for *Pcif1* knockout-induced osteoporosis. Prior research has identified *RSPO2* mutations in fetuses exhibiting severe limb disorders, emphasizing its critical role in skeletal development [71]. Conditional *Rspo2* ablation in *Osteocalcin*^+^ cells could lead to decreased body size and femoral trabecular bone in either male or female mice [72]. Overexpression of *Rspo2* promotes BMP-induced osteoblast mineralization *in vitro* [65]. *In vitro* and *in vivo* functional studies revealed administration of rhRSPO2 partially rescued the bone formation disruption and the restrained osteoblast differentiation of MSCs. The partial rescue suggests that PCIF1 may affect other signaling cascades related to bone biology. For example, RNA-seq results indicates that *Pcif1* deletion also downregulates the Calcium signaling and cGMP–PKG pathways. Besides, *Pcif1* deletion reduces the expression of multiple genes related to osteoblast differentiation and mineralization, e.g., *Bmp4*, *Bmp5*, *Alpl*, and *Sp7*. Moreover, RSPO2 efficacy is likely influenced by the abundance of downstream receptors and WNT ligands [46]. It is also possible that some developmental defects are not fully reversible when established during the early postnatal period in *Pcif1*-deficient mice.

It should be noted that osteoporosis in humans is predominantly an age-related condition, whereas our study primarily employed young mouse models (3- and 6-week-old). Future studies using aged or osteoporotic animal models will be essential to determine whether PCIF1-mediated m^6^Am modification similarly contributes to age-associated bone loss.

In sum, our findings demonstrate PCIF1 as a candidate associated with osteoporosis-related traits. *Pcif1* deletion impairs trabecular bone formation by suppressing osteogenic differentiation. Mechanically, PCIF1-mediated m^6^Am modulation enhances the mRNA stability of Wnt-related genes. Our findings unveil a pivotal role of PCIF1-mediated m^6^Am modulation in skeletal homeostasis, highlighting its potential for therapeutic intervention.

## Methods

### Genome-wide association studies (GWAS)

Data were obtained from the GEnetic Factors for Osteoporosis (GEFOS) consortium (http://www.gefos.org/). BMD assessments in studies like GEFOS2, GEFOS Life Course, and the Louisiana Osteoporosis Study (LOS) utilized dual-energy X-ray absorptiometry (DXA). The UK Biobank studies (UKBB), including UKBB2017 and UKBB2018, estimated BMD using quantitative heel ultrasound (eBMD). Fracture data were sourced from GEFOS2 ALLFX and the UKBB studies, identified through hospital-based diagnoses (ICD10 codes) or self-reported incidents via questionnaires. Participants reported fractures occurring within the past 5 years, with exclusions for fractures of the skull, face, hands, and feet, as well as pathological fractures due to malignancy, atypical femoral fractures, periprosthetic fractures, or healed fractures. For detailed information on the GWAS datasets, please refer to S1 Table.

We conducted gene-based association tests using the R package “snpsettest,” which aggregates SNP effects while accounting for linkage disequilibrium (LD) between markers [73]. This approach incorporates LD among SNPs within each gene and employs permutation-based simulation to compute gene-based *p*-values. We evaluated association signals from all SNPs within a 50kb window before and after the gene for SNP mapping. To infer pairwise LD correlations among SNPs, we utilized 1000 Genomes data [74] (available at https://www.cog-genomics.org/plink/2.0/resources) aligned to human reference genomes such as Hg18, Hg19, or Hg38, selected based on the GWAS studies. Selection criteria considered race and sex to accurately estimate LD. Additionally, we utilized the UCSC Genome Browser’s UCSC Overlap Lift (UCSC’s USCS overlift) tool to convert coordinates from the Hg19 human reference genome to the Hg18 reference genome. A gene with *p*-values less than 0.05 was considered significant.

To assess the regulatory relevance of variants identified from the gene-based association analyses, we performed systematic variant annotation and eQTL mapping. Lead and nominally significant SNPs (*p* ≤ 0.05) for PCIF1 were examined. Variant annotation was conducted using the R package biomaRt [75], which queried the Ensembl database [76] to retrieve genomic coordinates, variant consequences, and gene annotations for the top SNPs identified from the gene-based analyses. The annotation was performed according to the genome reference version used in the corresponding GWAS datasets (S1 Table). This process enabled classification of variants as coding or noncoding and identification of potential regulatory regions proximal to each gene.

Significant single-tissue eQTL data were obtained from the GTEx v8 database [77] using the R package gtexr [78] [https://ropensci.r-universe.dev/gtexr]. Gene symbols were first mapped to their corresponding Ensembl identifiers, and all significant variant–gene–tissue associations were retrieved across GTEx tissues. These eQTLs were then intersected with the top SNPs with matched rsID identified from the gene-based analyses to identify overlapping regulatory variants. For each overlap, we recorded the corresponding tissue, the pre-computed eQTL association *p*-value, and normalized effect size (NES) as provided by the original eQTL analysis [77].

### Transcriptome-wide association studies (TWAS)

To identify causal genes and variants, webTWAS integrates seven statistical models from three widely used TWAS software packages: PrediXcan [79]/S-PrediXcan [80], TWAS-FUSION (best-TWAS, BLUP, LASSO, Top1) [81], and UTMOST (joint tissue GBJ model) [82]. Each gene is analyzed across 47 GTEx tissues, generating 47 × 6 + 1 association results, with UTMOST combining results into a single score. The 1000 Genomes Project LD matrix is applied across all models, and a Bonferroni-corrected significance threshold accounts for multiple testing.

Given the tissue-specific nature of gene expression, webTWAS employs tissue-specific enrichment analysis based on TSEA-DB [83] to identify disease-relevant tissues. We used webTWAS to examine the “PCIF1” gene in relation to osteoporosis-related traits, including osteoporosis, osteopenia, fractures, and other bone density disorders. This analysis helped identify genes potentially involved in bone health and osteoporosis pathogenesis.

### Subjects and Sample Collection for snRNA-seq analysis

Femoral head tissues were collected from 14 patients undergoing total hip arthroplasty (THA) at Tulane Lakeside Hospital (Metairie, LA, USA) between 2023 and 2024. The sample included individuals aged 42–78 years (mean ± SD: 62.8 ± 11.4), consisting of 6 females, 7 males, and 1 individual with missing sex information. The majority were White (64.3%), followed by African American (28.6%) and Hispanic (7.1%). Detailed sample characteristics are provided in S2 and S3 Tables. THA is a routine orthopedic procedure that provides access to high-quality human bone tissue, which is typically discarded during surgery. Although all participants were diagnosed with osteoarthritis—a condition highly prevalent in the aging population that primarily affects the joint surface—it has limited impact on the underlying trabecular bone, as described in previous studies [30]. Thus, femoral head specimens obtained through THA serve as a practical and ethically appropriate source of bone tissue for investigating age-related skeletal biology. Patients with chronic conditions known to affect bone metabolism—including hepatic or renal dysfunction, thyroid or parathyroid disorders, diabetes, malignancies, malabsorption syndromes, hematologic diseases, or a history of pathological fractures—were excluded. The study protocol was approved by the Tulane University Institutional Review Board (Tulane IRB #: 2022-530), and written informed consent was obtained from all participants. All procedures involving human participants were conducted in accordance with the principles of the Declaration of Helsinki.

### Isolation of osteolineage cells from human femoral head tissue

Upon surgical removal, femoral heads were immediately placed in pre-chilled 1× phosphate-buffered saline (PBS) at 4 °C and transported to the Tulane Center for Biomedical Informatica and Genomics laboratory on ice. All subsequent procedures were performed under a biosafety level 2 cabinet, with instruments and surfaces sterilized using 75% ethanol. Residual soft tissue was removed, and bone specimens were cut into approximately 5 mm^3^ fragments. These fragments were thoroughly rinsed with PBS until the supernatant was free of blood and then temporarily stored in ice-cold alpha minimum essential medium (α-MEM). For enzymatic digestion, bone fragments were incubated in a digestion buffer containing 0.1% collagenase type II (Worthington), DNase I (Sigma), 10% fetal bovine serum (FBS), and antibiotics at 37 °C for 3 hours in a water bath with gentle mixing every 30 min. The resulting cell suspension was filtered through a 40 μm cell strainer and centrifuged. RBCs were lysed using RBC lysis buffer (e.g., BioLegend), followed by a second centrifugation. Cell viability and concentration were assessed using acridine orange/propidium iodide (AOPI) staining and quantified with an automated cell counter. Only samples containing more than 1 × 10⁶ total cells with viability greater than 80% were selected for downstream sorting.

To enrich for osteolineage cells, hematopoietic and immune-derived cells were removed using magnetic-activated cell sorting (MACS; Miltenyi Biotec). First, CD45⁺ cells were depleted by incubating 1 × 10⁷ total cells with 20 μL of CD45 MicroBeads and 80 μL of pre-chilled MACS buffer for 15 min on ice. The cell suspension was then passed through an LS column placed in a SuperMACS II separator, and the flow-through containing CD45⁻ cells was collected. These CD45⁻ cells were subsequently incubated with CD43 MicroBeads under the same conditions and processed through a second LS column to remove CD43⁺ cells. The resulting CD45⁻CD43⁻ cell population was collected and used for downstream scRNA-seq. The total cell count and viability of the sorted CD45⁻CD43⁻ population were reassessed using AOPI staining, performed by mixing 10 μL of chilled AOPI stain with 10 μL of the cell suspension and measured under the “Cell line, viability propidium iodide” setting, using a dilution factor of 2. Samples yielding more than 3 × 10⁵ cells with viability greater than 80% were fixed by adding 100 μL of pre-warmed Enhancer and 275 μL of 50% glycerol to 1 mL of chilled Quenching Buffer. The mixture was gently pipetted until homogeneous and stored at −80 °C for long-term preservation, for up to 6 months.

### Single-cell RNA sequencing library preparation and data processing library preparation and sequencing

scRNA-seq libraries were prepared using the 10× Genomics Chromium Single Cell 3′ Library & Gel Bead Kit v3 (Cat. No. 1000075). Approximately 5,000–10,000 sorted CD45⁻ cells per sample were loaded into the Chromium Controller to generate gel bead-in-emulsion (GEM) droplets, enabling cell lysis and reverse transcription of mRNA within nanoliter compartments. Cell viability (>85%) was confirmed using trypan blue staining prior to GEM generation. Following cDNA amplification, sequencing libraries were constructed according to the manufacturer’s protocol and quantified using Qubit and Bioanalyzer. Libraries were sequenced on the Illumina NovaSeq 6000 platform using paired-end 150 bp reads (PE150), with a target depth of at least 100,000 reads per cell to ensure robust transcriptome coverage.

### scRNA-seq data processing, annotation, and pathway analysis

scRNA-seq data were demultiplexed, aligned, and count matrices were generated using Cell Ranger v6.1.2 [84] aligning to GRCh38 with intronic regions included. Cell Ranger matrices were directly imported into Seurat v4. Cells [85] with 300–10,000 unique genes expressed, less than 10,000 unique UMIs, and less than 10% mitochondrial reads were retained in the final dataset. Individual samples were processed by log-normalization; the top 2,000 features were identified; the data was scaled; a principal component analysis (PCA) was computed, and the data was clustered via Louvain clustering using the top 30 principal components (PCs). We used DoubletFinder [86] to remove doublets on a per-sample basis using the top 30 PCs, an expected doublet rate equal to the number of cells in the sample or 125,000 cells (a default scaling factor), and other parameters remained as default. Doublet cells were removed, and samples were combined without computational batch correction. The full dataset was processed as above and visualized using UMAP with the top 30 PCs. Differentially expressed genes in each cluster were identified using the FindAllMarkers function in Seurat v4 with the Wilcoxon rank-sum test downsampled to a maximum of 1,000 cells per cluster, and these results were recorded in S1 Fig. Differentiation states of MSCs lineage were inferred using CytoTRACE (v0.3.3) [87] with default parameters using the log-normalized counts. To reconstruct the continuous differentiation trajectory from MSCs to mature osteoblasts (Mature_OB), we applied Monocle 2 (v2.4.0) [88]. The integrated Seurat object was converted into a CellDataSet object using the SeuratWrappers package, maintaining gene expression and metadata integrity. Genes used for ordering were selected based on differential expression across clusters, and dimensionality reduction was performed using DDRTree algorithm included in Monocle 2.

### Generation of global and conditional *Pcif1* knockout mice

For global knockout, the *Pcif1*^−/−^ mice were generated by targeting at the exon 5, 6, and 7 of *Pcif1* gene [16]. For conditional knockout, *Pcif1*^*fl/+*^ mice were generated by inserting dual loxP sites targeting exon 5, 6, and 7 [16]. MSC-specific *Pcif1* knockout mice were obtained by mating *Pcif1*^*fl/+*^ mice with *Prrx1-Cre* mice (The Jackson Laboratory). Myeloid cell-specific *Pcif1* knockout mice were achieved by crossing *Pcif1*^*fl/+*^ mice with *LysM-Cre* mice (The Jackson Laboratory). *Prrx1-Cre;Pcif1*^*fl/fl*^ mice aged 3 weeks received weekly intraperitoneal injection of the WNT agonist rhRSPO2 (4 mg/kg) for five weeks and then sacrificed for subsequent examinations. All mice used in the study had a C57BL/6J genetic background, and were housed in SPF facilities following a 12-hour light-dark cycle (Animal Care Center of State Key Laboratory of Oral Diseases). All animal experiments were performed in compliance with institutional animal care and ethical guidelines (Research Ethics Committee of West China Hospital of Stomatology).

### Micro-CT and histomorphometric analysis

Femurs isolated from 3- or 6-week-old mice were fixed before scanned by a μCT 80 cabinet micro-CT scanner (Scanco Medical, Switzerland). The femurs were imaged with a spatial resolution of 8 μm (70 kV, 200 mA, 0.5 mm aluminum filter, 300 ms integration time). The bone parameters were measured using the SCANCO Evaluation software (version 1.1.19.0, Scanco Medical). Visualization of trabecular and cortical bone was conducted using SCANCO Visualizer software (version 1.1.18.0, Scanco Medical).

For histomorphometric evaluation, decalcified samples were prepared as five-μm-thick sections for H&E staining (Solarbio, China) and TRAP staining (Solarbio, China). For Von Kossa staining and calcein double-labeling experiment, undecalcified femurs were prepared as ten-μm-thick sections using a Leica CM3050 S cryostat (Leica Biosystems, USA). All parameters of trabecular bone of distal femurs were calculated by OsteoMeasure software (OsteoMetrics, USA).

### Cell culture and mineralization assay

Murine MSCs were extracted from femurs of 6-week-old mice, and cultured in α-MEM medium (Gibco, USA) supplemented with 10% FBS, 100 units/mL penicillin and 100 μg/mL streptomycin (all from Gibco, USA). For osteogenic induction, the osteogenic medium was supplemented with 50 μg/mL ascorbic acid, 5 mM β-glycerophosphate, and 100 nM dexamethasone (all from Sigma), in addition to the original culture medium.

MSCs treated with osteogenic medium for 7 days were fixed and stained with ALP staining kit (Beyotime, China). Quantitative ALP activity was measured according to the manufacturer’s protocols (Nanjing Jiancheng, China). For ARS staining, MSCs receiving osteogenic induction for 14 days were stained with 1% Alizarin red S (Solarbio, China). Quantification of mineralization was assessed through the OD value at 450 nm by a spectrophotometer (Thermo Fisher Scientific) after destained with 10% cetylpyridinium chloride.

Bone marrow cells were isolated and treated with ACK lysis buffer, and then cultured overnight at 37°C. Nonadherent BMDMs were collected and cultured in DMEM complete medium (Gibco, USA) supplemented with 50 ng/mL M-CSF and 50 ng/mL RANKL (R&D Systems, USA) for 5 days.

### Quantitative RT-PCR and western blot

RNA was extracted from MSCs after a 7-day osteogenic induction by Trizol reagent (Invitrogen, USA) and treated with a cDNA Synthesis kit following the instructions (Yeasen, China). The expression of each gene was measured using a 2^−ΔΔCt^ method and normalized to *Gapdh* (Yeasen, China). Proteins were obtained by a total protein extraction kit (Cell signaling, USA), and then subjected to western blot analyses. The primers and antibodies used in the manuscript were listed in S4 and S5 Tables.

### Bulk RNA sequencing

MSCs from *Prrx1-Cre;Pcif1*^*fl/fl*^ mice and *Pcif1*^*fl/fl*^ littermates were cultured in osteogenic medium for 7 days. Total RNAs were extracted by Trizol reagent (Invitrogen, USA) and purified by using poly-T oligo-attached magnetic beads. Sequencing libraries were generated and subjected to Illumina PE150. Clean data was processed and mapped to the Mus musculus reference genome (mm10) by HISAT2. Differentially expressed genes were selected by DESeq2 R package with |fold change| ≥ 2.0 and padj ≤ 0.05. GO and KEGG enrichment analyses were performed using clusterProfiler R package.

### m^6^Am-Exo-Seq analysis

m^6^Am-Exo-Seq datasets were obtained from the published database GSE151229, and mapped to the Mus musculus reference genome (mm10). m^6^Am peaks of *Wnt11*, *Fzd4* and *Fgfr2* were visualized using IGV software.

### m6AmPred prediction

Predictions of m^6^Am modification sites were performed using the m6AmPred website (http://180.208.58.19/m6ampred/index.html) [42]. The sequences of mouse *Wnt11*, *Fzd4*, and *Fgfr2* transcripts were retrieved from the National Center for Biotechnology Information (NCBI) database. The calculated likelihood ratio is capped at 99, and the higher values indicate the greater possibility. The candidate m^6^Am sites within 500 nt downstream of the TSS were selected for further analyses.

### MeRIP-qPCR

The enrichment of m^6^Am-modified RNA fragments was performed using the EpiQuik CUT&RUN m^6^A RNA Enrichment (MeRIP) Kit (EpigenTek, USA) according to the manufacturer’s instructions. Total RNA of MSCs was extracted and subjected to enrichment procedures. RNA fragments containing m^6^Am were captured, purified and reverse transcribed followed by RT-qPCR using site-specific primers. The relative m^6^Am enrichment levels were calculated as the percentage of the m^6^Am signal in the IP group compared to that in the IgG control.

### RNA decay assay

MSCs from *Prrx1-Cre;Pcif1*^*fl/fl*^ mice and *Pcif1*^*fl/fl*^ littermates were seeded at the concentration of 300 thousand per well and subjected to osteogenic induction for 7 days. Then, the cells were treated with actinomycin D (10 μg/mL) for 0, 2, 4, and 6 hours. Total RNA was harvested at pre-determined time points for qRT-PCR analyses. Gene expression was normalized to *t = 0*.

### Dual-luciferase reporter assay

293T cells were seeded in 24-well plates and co-transfected with 500 ng of the reporter plasmid and PCIF1 siRNA, along with a PCIF1 overexpression plasmid or a catalytically inactive PCIF1 mutant plasmid. The PCIF1 overexpression plasmid and PCIF1 mutant plasmid (aa160–163, LFPD to AFPA) were constructed as reported [13,15]. After 48 h, Firefly and Renilla luciferase activities were measured using the Dual-Luciferase Reporter Assay System (Promega, USA). Firefly luciferase activity was normalized to Renilla.

### Statistical analysis

All data were expressed as mean ± SD. Comparison between two independent groups was performed using an unpaired two-tailed Student *t* test. Comparisons among multiple groups (i.e., more than two groups) were conducted using one-way ANOVA, followed by Tukey’s post hoc test for pairwise comparisons. A *p*-value below 0.05 was regarded as statistically significant.

## Supporting information

S1 Fig Heatmap showing normalized gene expression, scaled by row (gene), for key cell lineage marker genes across the corresponding cell clusters. (TIF)

S2 Fig Global deletion of *Pcif1* decreases bone mass of female mice. **(A)** Representative Micro-CT images of femurs from 6-week-old female *Pcif1*^*−/−*^ knockout mice and *Pcif1*^*+/+*^ littermates. Scale bar, 200 μm. **(B)** Quantitative analyses of distal trabecular bone and midshaft cortical thickness of femurs (*n* = 8). **(C)** Representative images of Von Kossa staining of undecalcified femoral sections. Scale bar, 500 μm. **(D)** Representative TRAP images of trabecular bone from the femoral metaphysis. Scale bar, 50 μm. **(E)** Quantitation of osteoblast and osteoclast numbers of trabecular bone (*n* = 8). **(F)** Representative images demonstrating calcein double-labeling patterns in trabecular bone from the femoral metaphysis. Scale bar, 20 μm. **(G)** Quantitation of mineralization apposition rate (MAR) and bone formation rate (BFR/BS) of trabecular bone (*n* = 8). The data underlying panels B, E, and G can be found in S1 Data (Sheet S2 Fig).
(TIF)

S3 Fig *LysM*-driven *Pcif1* knockout does not cause changes of bone mass of female mice. **(A)** Representative Micro-CT images of femurs from 6-week-old female *LysM-Cre;Pcif1*^*fl/fl*^ mice and *Pcif1*^*fl/fl*^ littermates. Scale bar, 200 μm. **(B)** Quantitation of bone parameters (*n* = 8). The data underlying panel B can be found in S1 Data (Sheet S3 Fig).
(TIF)

S4 Fig Bulk RNA-seq and m^6^Am-exo-seq. **(A)** Volcano plot showing differentially expressed genes. **(B)** qRT-PCR analysis of *Wnt11*, *Fzd4*, and *Fgfr2* in MSCs cultured *in vitro* without osteogenic induction (*n* = 3). **(C)** Diagram of integrative analysis. The data underlying panel B can be found in S1 Data (Sheet S4 Fig).
(TIF)

S1 Table GWAS summary statistics data source. (PDF)

S2 Table Donor Metadata for scRNA analysis. (PDF)

S3 Table Sample characteristics stratified by sex (with missing category). (PDF)

S4 Table Antibodies used in the manuscript. (PDF)

S5 Table Primers used in the manuscript. (PDF)

S1 File Bone-related SNPs for m^6^Am regulators. (XLSX)

S2 File GTEx eQTL outputs of m^6^Am regulators. (XLSX)

S3 File TWAS analysis for *PCIF1.* (XLSX)

S4 File m6AmPred results for *Wnt11.* (CSV)

S5 File m6AmPred results for *Fzd4.* (CSV)

S6 File m6AmPred results for *Fgfr2.* (CSV)

S1 Raw Images Unedited blot and gel images. (PDF)

S1 Data Excel file with all individual numerical values corresponding to the data presented in the main and supporting figures. (XLSX)

## Abbreviations

ALP: alkaline phosphatase
AOPI: acridine orange/propidium iodide
ARS: alizarin red staining
BFR/BS: bone formation rate
BMD: bone mineral density
eBMD: estimated bone mineral density
FBS: fetal bovine serum
GEM: gel bead-in-emulsion
GO: gene ontology
GWAS: genome-wide association studies
KEGG: Kyoto Encyclopedia of Genes and Genomes
LD: linkage disequilibrium
MAR: mineral apposition rate
MSCs: mesenchymal stem cells
NCBI: National Center for Biotechnology Information
OB: osteoblast precursors
PBS: phosphate-buffered saline
PCF11: Premature Cleavage Factor II
PCIF1: Phosphorylated CTD Interacting Factor 1
PCs: principal components
RBCs: red blood cells
RSPO2: R-spondin-2
scRNA-seq: single-cell RNA sequencing
THA: total hip arthroplasty
TRAP: tartrate-resistant acid phosphatase
TSS: transcription start sites
TWAS: transcriptome-wide association studies
UMIs: unique molecular identifiers
α-MEM: alpha minimum essential medium
