# Supplementary material for: The m6Am methyltransferase PCIF1 promotes osteogenic differentiation of mesenchymal stem cells through stabilization of Wnt-related transcripts
Source: PLoS Biol. 2026 Apr 6;24(4):e3003739. doi: 10.1371/journal.pbio.3003739 (PMC13068325; doi:10.1371/journal.pbio.3003739)
Supplement: S1 Table — (PDF) [file pbio.3003739.s005.pdf]

S1 Table. GWAS summary statistics data source

Short Study Name	Traits	Title and note	PMID
GEFOS2	FN-BMD<br>LS-BMD	Genome-wide meta-analysis identifies 56 bone mineral density loci and reveals 14 loci associated with risk of fracture (1).<br>Provided both pooled and age-stratified summary statistics.	22504420
GEFOS 2015	FA-BMD<br>FN-BMD<br>LS-BMD	Whole-genome sequencing identifies EN1 as a determinant of bone density and fracture. Nature 2015 (2).	26367794
GEFOS 2017	Total body less head BMD and Lean Mass	Bivariate genome-wide association meta-analysis of pediatric musculoskeletal traits reveals pleiotropic effects at the SREBF1/TOM1L2 locus (3)	28743860
UKBB 2017	eBMD	Identification of 153 new loci associated with heel bone mineral density and functional involvement of GPC6 in osteoporosis (4)	28869591
GEFOS LIFE COURSE	Total body BMD	Life-Course Genome-wide Association Study Meta-analysis of Total Body BMD and Assessment of Age-Specific Effects (5)<br>Provided both pooled and age-stratified summary statistics. Five age strata, each spanning 15 years, including “less 15”, “15 to 30”, “30 to 45”, “45 to 60”, and “60 more”.	29304378
UKBB 2018	eBMD<br>Fracture	An atlas of genetic influences on osteoporosis in humans and mice (6)<br>The p-values from infinitesimal model were used for fracture data, and the p-values from non-infinitesimal model were used for eBMD data.	30598549
Estrogen_2018	Estradiol and Estrone	Genetic Determinants of Circulating Estrogen Levels and Evidence of a Causal Effect of Estradiol on Bone Density in Men (7)	29325096
GEFOS ALLFX	Fracture	Assessment of the genetic and clinical determinants of fracture risk: genome wide association and mendelian randomization study (8)	30158200
Skull BMD	Skull BMD	Genome Wide Association Metanalysis of Skull Bone Mineral Density Identifies Loci Relevant For Osteoporosis And Craniosynostosis (9)	37402774

UKBB Falls	Falls	Genetic basis of falling risk susceptibility in the UK Biobank Study (10)	32999390
LOS	FN-BMD<br>LS-BMD	A multiethnic whole genome sequencing study to identify novel loci for bone mineral density (11)	34673960

References

1. K. Estrada *et al.*, Genome-wide meta-analysis identifies 56 bone mineral density loci and reveals 14 loci associated with risk of fracture. *Nat Genet* **44**, 491-501 (2012).
2. H. F. Zheng *et al.*, Whole-genome sequencing identifies EN1 as a determinant of bone density and fracture. *Nature* **526**, 112-117 (2015).
3. C. Medina-Gomez *et al.*, Bivariate genome-wide association meta-analysis of pediatric musculoskeletal traits reveals pleiotropic effects at the SREBF1/TOM1L2 locus. *Nat Commun* **8**, 121 (2017).
4. J. P. Kemp *et al.*, Identification of 153 new loci associated with heel bone mineral density and functional involvement of GPC6 in osteoporosis. *Nat Genet* **49**, 1468-1475 (2017).
5. C. Medina-Gomez *et al.*, Life-Course Genome-wide Association Study Meta-analysis of Total Body BMD and Assessment of Age-Specific Effects. *Am J Hum Genet* **102**, 88-102 (2018).
6. J. A. Morris *et al.*, An atlas of genetic influences on osteoporosis in humans and mice. *Nat Genet* **51**, 258-266 (2019).
7. A. L. Eriksson *et al.*, Genetic Determinants of Circulating Estrogen Levels and Evidence of a Causal Effect of Estradiol on Bone Density in Men. *J Clin Endocrinol Metab* **103**, 991-1004 (2018).
8. K. Trajanoska *et al.*, Assessment of the genetic and clinical determinants of fracture risk: genome wide association and mendelian randomisation study. *BMJ* **362**, k3225 (2018).
9. C. Medina-Gomez *et al.*, Bone mineral density loci specific to the skull portray potential pleiotropic effects on craniosynostosis. *Commun Biol* **6**, 691 (2023).
10. K. Trajanoska *et al.*, Genetic basis of falling risk susceptibility in the UK Biobank Study. *Commun Biol* **3**, 543 (2020).
11. J. Greenbaum *et al.*, A multiethnic whole genome sequencing study to identify novel loci for bone mineral density. *Hum Mol Genet* **31**, 1067-1081 (2022).
