# Supplementary material for: The m6Am methyltransferase PCIF1 promotes osteogenic differentiation of mesenchymal stem cells through stabilization of Wnt-related transcripts
Source: PLoS Biol. 2026 Apr 6;24(4):e3003739. doi: 10.1371/journal.pbio.3003739 (PMC13068325; doi:10.1371/journal.pbio.3003739)
Supplement: S2 Table — (PDF) [file pbio.3003739.s006.pdf]

S2 Table. Donor Metadata for scRNA analysis

Sample ID	Race	Gender	Age	Diagnosis	Date[1]
Sample 24	Caucasian	Female	55	Osteoarthritis	9/20/23
Sample 36	Caucasian	Female	76	Osteoarthritis	12/4/23
Sample 65	Caucasian	Female	50	Osteoarthritis	7/15/24
Sample 74	Caucasian	Female	71	Osteoarthritis	9/25/24
Sample 77	Caucasian	Female	73	Osteoarthritis	11/29/24
Sample43	Caucasian	Female	78	Osteoarthritis	2/12/24
Sample 25	African-American	Male	42	Osteoarthritis	9/25/23
Sample 30	African-American	Male	57	Osteoarthritis	10/23/23
Sample 63	African-American	Male	65	Osteoarthritis	6/26/24
Sample 71	African-American	Male	57	Osteoarthritis	9/5/24
Sample 29	Caucasian	Male	54	Osteoarthritis	10/16/23
Sample 52	Caucasian	Male	62	Osteoarthritis	4/29/24
Sample 73	Caucasian	Male	76	Osteoarthritis	9/18/24
Sample 32	Hispanic	NA	NA	Osteoarthritis	11/6/23

1: Sample collection date
