# Supplementary material for: The m6Am methyltransferase PCIF1 promotes osteogenic differentiation of mesenchymal stem cells through stabilization of Wnt-related transcripts
Source: PLoS Biol. 2026 Apr 6;24(4):e3003739. doi: 10.1371/journal.pbio.3003739 (PMC13068325; doi:10.1371/journal.pbio.3003739)
Supplement: S3 Table — (PDF) [file pbio.3003739.s007.pdf]

S3 Table. Sample characteristics stratified by sex (with missing category)

	Level	Overall	Female	Male	NA[1]
n		14	6	7	1
Race (%)	African-American	4 (28.6)	0 (0.0)	4 (57.1)	0 (0.0)
	Caucasian	9 (64.3)	6 (100.0)	3 (42.9)	0 (0.0)
	Hispanic	1 (7.1)	0 (0.0)	0 (0.0)	1 (100.0)
Age (mean (SD))		62.77 (11.40)	67.17 (11.72)	59.00 (10.46)	NA

1: Uknown sex information
