# Supplementary material for: The m6Am methyltransferase PCIF1 promotes osteogenic differentiation of mesenchymal stem cells through stabilization of Wnt-related transcripts
Source: PLoS Biol. 2026 Apr 6;24(4):e3003739. doi: 10.1371/journal.pbio.3003739 (PMC13068325; doi:10.1371/journal.pbio.3003739)
Supplement: S4 Table — (PDF) [file pbio.3003739.s008.pdf]

S4 Table. Antibodies used in the manuscript

**Antibodies**	**Source**	**Identifier**
Anti-PCIF1 antibody produced in rabbit	Sigma-Aldrich	Cat# HPA049517
Anti-α-Tubulin antibody (Rabbit mAb)	Huabio	Cat# HA721914
Anti-rabbit IgG, HRP-linked Antibody (Goat pAb)	Huabio	Cat# HA1001
Anti-FGFR2 antibody produced in rabbit	Proteintech	Cat# 84205-4-RR
Anti-WNT11 antibody (Rabbit mAb)	Abcam	Cat# ab316738
Anti-FZD4 antibody (Rabbit pAb)	Zenbio	Cat# 821422
Anti-β-Catenin antibody (Rabbit pAb)	Proteintech	Cat# 51067-2-AP
Anti-β-Catenin non-phospho(active) (Rabbit mAb)	Abcam	Cat# ab305261
Anti-β-Catenin antibody (Rabbit mAb)	Abcam	Cat# ab32572
