# Supplementary material for: The m6Am methyltransferase PCIF1 promotes osteogenic differentiation of mesenchymal stem cells through stabilization of Wnt-related transcripts
Source: PLoS Biol. 2026 Apr 6;24(4):e3003739. doi: 10.1371/journal.pbio.3003739 (PMC13068325; doi:10.1371/journal.pbio.3003739)
Supplement: S5 Table — (PDF) [file pbio.3003739.s009.pdf]

S5 Table. Primers used in the manuscript

Gene name	Application	Sequence
Pcif1 KO F	Genotyping	TGACTTGCTCGCTCCCTGTCTCTAT
Pcif1 KO R	Genotyping	CAATGCCTGTGGGACAAGAGAGCT
Pcif1 WT F	Genotyping	GTGTTGCTCAGCCTGCCTGTAATTG
Pcif1 WT R	Genotyping	CAATGCCTGTGGGACAAGAGAGCT
Pcif1-flox-1 F	Genotyping	TGGGGAATCCTGATCTGGGGAAGAA
Pcif1-flox-1 R	Genotyping	AGCTTCCTTCTTCCTGACTCCCTCA
Pcif1-flox-2 F	Genotyping	GTGTTGCTCAGCCTGCCTGTAATTG
Pcif1-flox-2 R	Genotyping	AGTTGCTTGGCAACAGAGGATCACA
Cre-1	Genotyping	ACTGGGATCTTCGAACTCTTTGGAC
Cre-2	Genotyping	GATGTTGGGGCACTGCTCATTCACC
Cre-3	Genotyping	CCATCTGCCACCAGCCAG
Cre-4	Genotyping	TCGCCATCTTCCAGCAGG
Pcif1 F	qPCR	GGCAACGGAGTGAAGAAG
Pcif1 R	qPCR	CAGCATTGGTCTGGATGTC
Gapdh F	qPCR	TGTGTCCGTCGTGGATCTGA
Gapdh R	qPCR	TTGCTGTTGAAGTCGCAGGAG
Dlx5 F	qPCR	CTGGCCGCTTTACAGAGAAG
Dlx5 R	qPCR	CTGGTGACTGTGGCGAGTTA
Bglap F	qPCR	TTGGTGCACACCTAGCAGAC
Bglap R	qPCR	ACCTTATTGCCCTCCTGCTT
Col1a1 F	qPCR	TAGGCCATTGTGTATGCAGC
Col1a1 R	qPCR	ACATGTTCAGCTTTGTGGACC
Runx2 F	qPCR	GGTACTTCGTCAGCATCCTATCAG
Runx2 R	qPCR	GCTTCCGTCAGCGTCAACAC
Alp F	qPCR	AACCCAGACACAAGCATTCC
Alp R	qPCR	GCCTTTGAGGTTTTTGGTCA
Sp7 F	qPCR	ATGGCGTCCTCTCTGCTTG
Sp7 R	qPCR	TGAAAGGTCAGCGTATGGCTT
Nfatc1 F	qPCR	GGAGAGTCCGAGAATCGAGAT
Nfatc1 R	qPCR	TTGCAGCTAGGAAGTACGTCT
Acp5 F	qPCR	CACTCCCACCCTGAGATTTGT
Acp5 R	qPCR	CATCGTCTGCACGGTTCTG
Ctsk F	qPCR	GAAGAAGACTCACCAGAAGCAG
Ctsk R	qPCR	TCCAGGTTATGGGCAGAGATT
Src F	qPCR	GAACCCGAGAGGGACCTTC
Src R	qPCR	GAGGCAGTAGGCACCTTTTGT
Fgfr2 F	qPCR	GCAAGGTTTACAGCGATGCC
Fgfr2 R	qPCR	CCAGCATCCATCTCCGTCAC
Wnt11 F	qPCR	AATCAGACGCAACACTGTAAAC
Wnt11 R	qPCR	CTCGATGGAGGAGCAGTTC
Fzd4 F	qPCR	CCTCGGCTACAACGTGACC

Fzd4 R	qPCR	TGCACATTGGCACATAAACAGA
Wnt11-IP-F1	MeRIP-qPCR	AGGAGAGAGCTCCGGAGAT
Wnt11-IP-R1	MeRIP-qPCR	AGCCGGGGATCCTGGCAACT
Wnt11-IP-F2	MeRIP-qPCR	AAGAAGCTATCCTCGCCGC
Wnt11-IP-R2	MeRIP-qPCR	AAAGACTTTGGGACGCTCTC
Fzd4-IP-F1	MeRIP-qPCR	AACAGCGCGGCGTAGAGT
Fzd4-IP-R1	MeRIP-qPCR	ATGGACTGCTAGGGTCATG
Fgfr2-IP-F1	MeRIP-qPCR	CCCTTCCTAAAGGAGGGT
Fgfr2-IP-R1	MeRIP-qPCR	GGAAGCAGCGGATGTTCG
Fgfr2-IP-F2	MeRIP-qPCR	GGGTGTCTCTTTGCGGCT
Fgfr2-IP-R2	MeRIP-qPCR	TGAAACCCGAGCCGTTTCC
