# Supplementary material for: The m6Am methyltransferase PCIF1 promotes osteogenic differentiation of mesenchymal stem cells through stabilization of Wnt-related transcripts
Source: PLoS Biol. 2026 Apr 6;24(4):e3003739. doi: 10.1371/journal.pbio.3003739 (PMC13068325; doi:10.1371/journal.pbio.3003739)

# Unedited blot and gel images

## Figure 5D-FGFR2

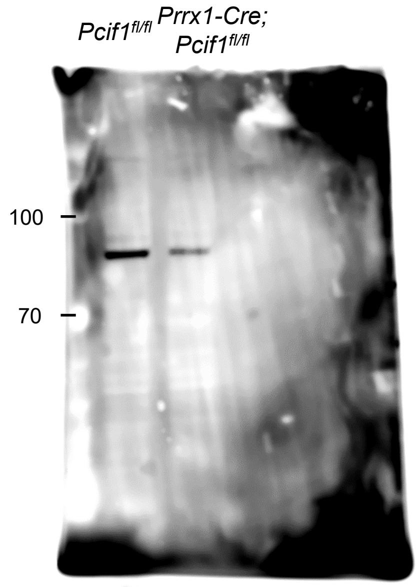


## Figure 5D-WNT11

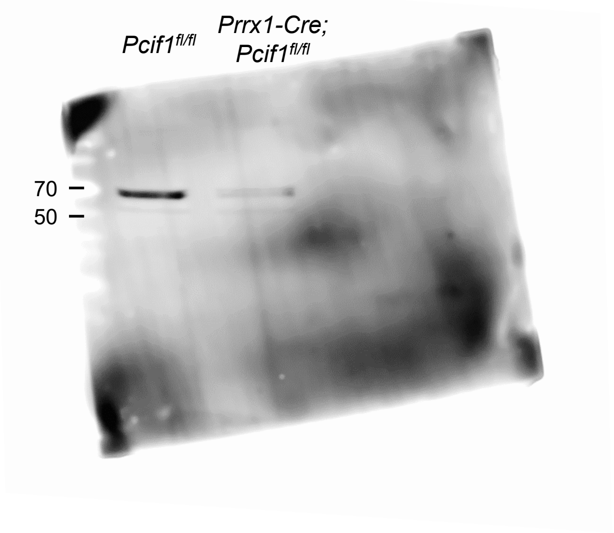


## Figure 5D-FZD4

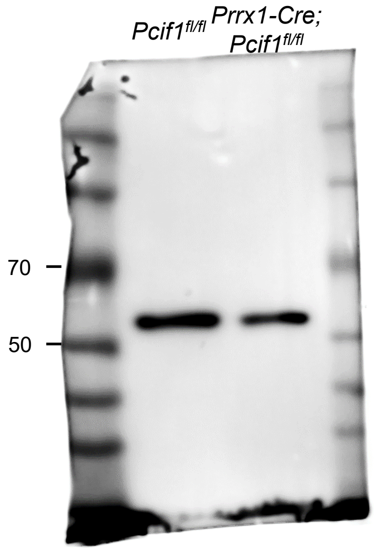


## Figure 5D-β-Catenin

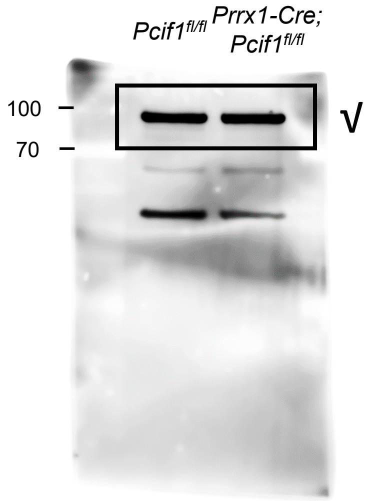


## Figure 5D-Active β-Catenin

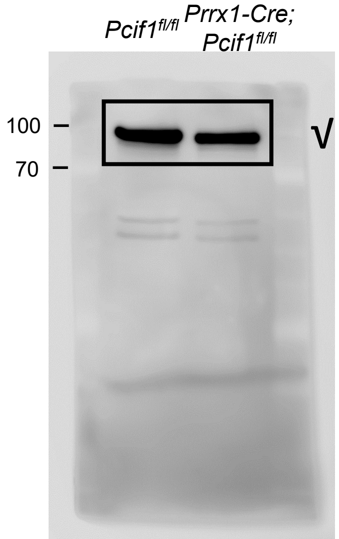


## Figure 5D-α-Tub

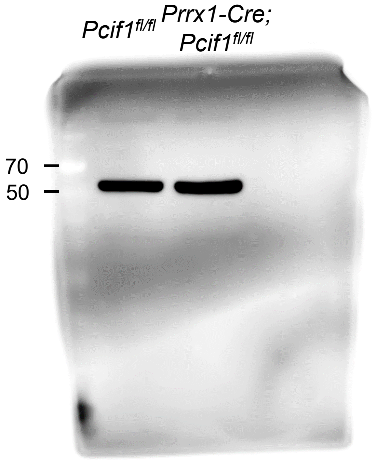

# Figure 7C-FGFR2

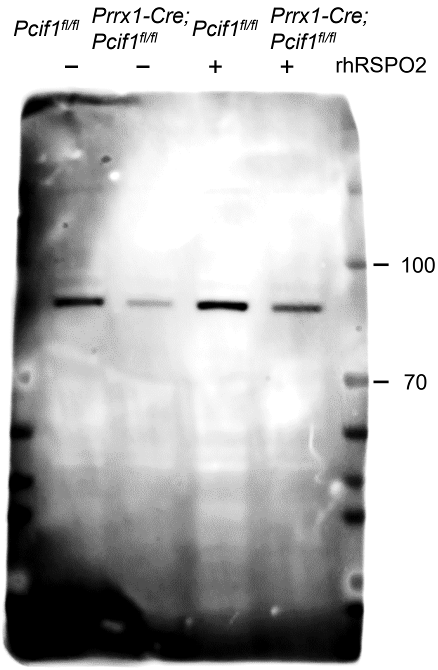


# Figure 7C-WNT11

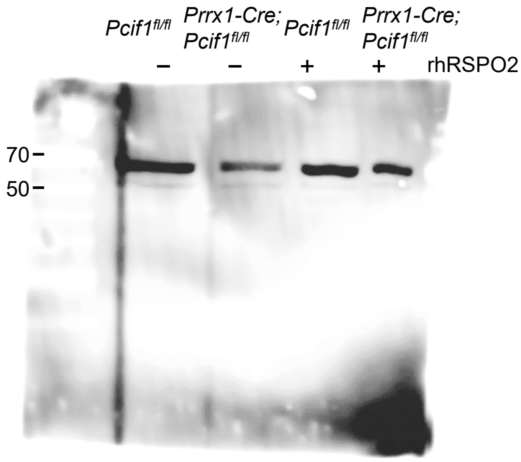


# Figure 7C-FZD4

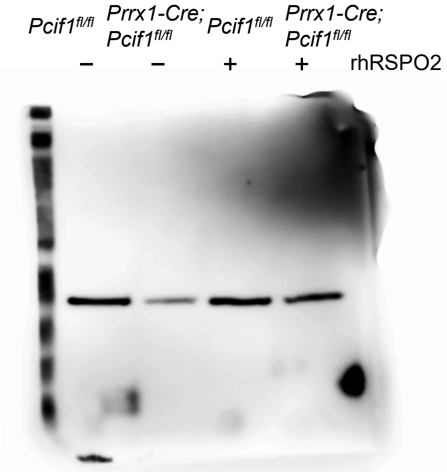


Sample-only

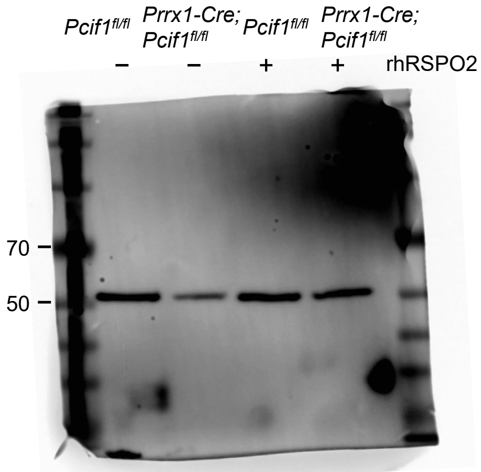


Marker+Sample-merged

# Figure 7C-β-Catenin

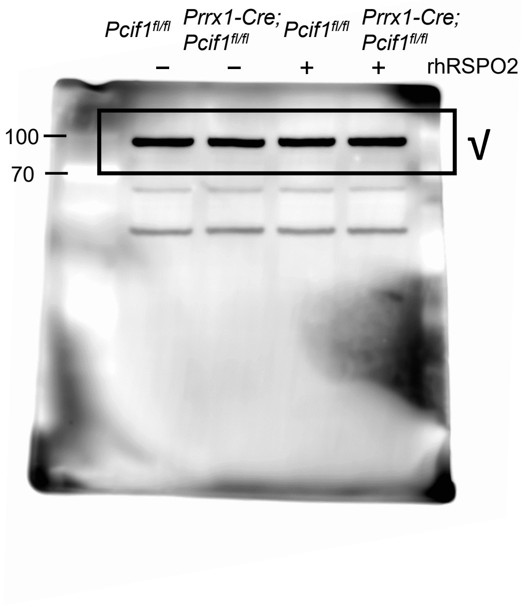


# Figure 7C-Active β-Catenin

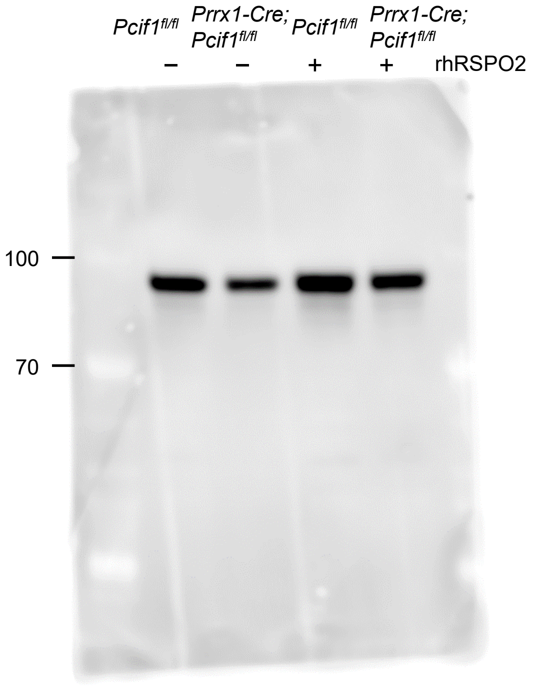


# Figure 7C-α-Tub

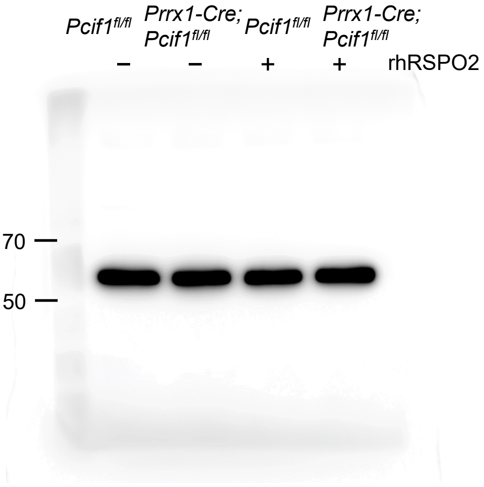

Supplement: S1 Raw Images — (PDF) [file pbio.3003739.s016.pdf]
